# The Role of the Equine Herpesvirus Type 1 (EHV-1) US3-Encoded Protein Kinase in Actin Reorganization and Nuclear Egress

**DOI:** 10.3390/v8100275

**Published:** 2016-10-12

**Authors:** Alexandra Proft, Bart Spiesschaert, Satoko Izume, Selina Taferner, Maik J. Lehmann, Walid Azab

**Affiliations:** 1 Institut für Virologie, Robert von Ostertag-Haus, Zentrum für Infektionsmedizin, Freie Universität Berlin, Robert-von-Ostertag-Str. 7-13, 14163 Berlin, Germany; AlexProft@gmx.de (A.P.); bart.spiesschaert@fu-berlin.de (B.S.); selina@taferner.de (S.T.); 2 Department of Applied Veterinary Sciences, United Graduate School of Veterinary Sciences, Gifu University, 501-1193 Gifu, Japan; s5110002@edu.gifu-u.ac.jp; 3 Department of Life Sciences and Engineering, University of Applied Sciences Bingen, 55411 Bingen, Germany; mj.lehmann@th-bingen.de; 4 Department of Virology, Faculty of Veterinary Medicine, Zagazig University, 44519 Zagazig, Egypt

**Keywords:** US3, actin cytoskeleton, EHV-1, EHV-4, adhesion molecules, EM

## Abstract

The serine-threonine protein kinase encoded by *US3* gene (pUS3) of alphaherpesviruses was shown to modulate actin reorganization, cell-to-cell spread, and virus egress in a number of virus species. However, the role of the US3 orthologues of equine herpesvirus type 1 and 4 (EHV-1 and EHV-4) has not yet been studied. Here, we show that *US3* is not essential for virus replication in vitro. However, growth rates and plaque diameters of a *US3*-deleted EHV-1 and a mutant in which the catalytic active site was destroyed were significantly reduced when compared with parental and revertant viruses or a virus in which EHV-1 *US3* was replaced with the corresponding EHV-4 gene. The reduced plaque sizes were consistent with accumulation of primarily enveloped virions in the perinuclear space of the *US3*-negative EHV-1, a phenotype that was also rescued by the EHV-4 orthologue. Furthermore, actin stress fiber disassembly was significantly more pronounced in cells infected with parental EHV-1, revertant, or the recombinant EHV-1 expressing EHV-4 *US3*. Finally, we observed that deletion of *US3* in EHV-1 did not affect the expression of adhesion molecules on the surface of infected cells.

## 1. Introduction

The US3 protein (pUS3) is a unique and multifunctional serine/threonine protein kinase, which is conserved among all alphaherpesviruses, including equine herpesvirus type 1 and type 4 (EHV-1 and EHV-4) [1,2,3]. Both EHV-1 and EHV-4 encode for *US3* genes that have a similarity of 81.3% [4]. It has been reported that pUS3 plays a role in the reorganization of the actin cytoskeleton, promotes nuclear egress of newly assembled nucleocapsids through the nuclear membrane, phosphorylates different viral and cellular protein substrates, modulates the host immune response, and protects infected cells from apoptosis [5,6,7]. During infection, several alphaherpesviruses, including herpes simplex virus type 1 and type 2 (HSV-1 and HSV-2), varicella zoster virus (VZV), pseudorabies virus (PRV), bovine herpesvirus type 1 (BHV-1), and Marek’s disease virus (MDV) modulate the actin cytoskeleton at almost every step of virus replication, from entry to egress and direct cell-to-cell spread. These events are dependent mostly on the kinase activity of pUS3 [6,8,9,10]. For PRV, group A p21-activated kinases (PAKs) were found to be involved in the pUS3-induced modulation of the actin cytoskeleton and the migration of virus particles through cell projections, ultimately leading to infection of neighboring cells [11]. However, PAKs were shown to play a limited role in the anti-apoptotic activity of PRV US3 [12].

EHV-1 and EHV-4 are members of the genus *Varicellovirus* in the *Alphaherpesvirinae* subfamily [13,14]. Although closely related as reflected by extensive similarity of the amino acid sequences of the encoded proteins (55–96%), their pathogenic potential differs significantly [15]. Both viruses have the same initial site of entry, replicate initially in mucosal epithelial cells of the upper respiratory tract, and cause respiratory disease. Only EHV-1, however, can efficiently infect the cells of lymphoid tissues, peripheral blood mononuclear cells (PBMC), and endothelial cells (EC) of blood vessels in the central nervous system and pregnant uterus [14]. In contrast to EHV-1 infection, almost all infections with EHV-4 do not progress beyond the respiratory epithelia. The answer to the question of why these two viruses have such different replication properties in vivo despite their close relatedness must be related to differences in functions exerted by different viral proteins, possibly including pUS3. Recently, we showed that EHV-1 deleted for the *US3* gene lost its ability to transfer from infected PBMC to EC [16], but we still do not know the exact mechanism.

After herpesvirus entry and genome replication, nucleocapsids are assembled in the nucleus. Then, newly formed nucleocapsids egress from the nucleus by first budding at the inner leaflet of the nuclear membrane, where they acquire the first envelope. The primarily enveloped viruses fuse to the outer leaflet of the nuclear membrane and are released into the cytoplasm [17]. pUS3 of PRV and HSV-1 appear to have a role during this process as deletion of the *US3* gene resulted in the accumulation of virus particles in the perinuclear space and significantly reduced virus titers [18,19,20]. It has been shown that pUS3 is necessary for the homogenous distribution of the primary tegument and envelope proteins, pUL31 and pUL34, along the nuclear membrane, a process that is mediated by phosphorylation of both proteins [7,21].

In this report, we aimed to study the role of pUS3 in EHV-1 replication, cell-to-cell spread, and reorganization of actin cytoskeleton in host cells. We further investigated the role of pUS3 on virus egress from the nucleus. For this purpose, we constructed EHV-1-mutants with a point mutation targeting the catalytic activity site (EHV-1^D207A^) conserved among alphaherpesviruses [6], a deletion of the *US3* gene (EHV-1∆US3) and a recombinant EHV-1 harboring the EHV-4 *US3* gene in lieu of EHV-1 *US3* (EHV-1-US3_4).

## 2. Materials and Methods

### 2.1. Viruses and Cells

Infectious bacterial artificial chromosome (BAC) clones of EHV-1 strain Ab4 [22] and EHV-4 strain TH20p [23] were used in this study. Both BAC clones contain the green fluorescent protein (GFP) gene under the control of a cytomegalovirus (CMV) promoter, facilitating GFP expression in infected cells. Viruses were grown and titrated on equine dermal (ED) cells propagated in Iscove’s modified Dulbecco’s medium (IMDM, Invitrogen, Darmstadt, Germany) supplemented with 10% fetal bovine serum (FBS, Biochrom, Berlin, Germany), 100 U/mL penicillin, and 100 μg/mL streptomycin. Equine PBMC were isolated from heparinized blood collected from healthy horses by Biocoll-density gradient separation (Biochrom AG, Berlin, Germany), following the manufacturer’s instructions. After two washing steps, cells were suspended in Roswell Park Memorial Institute (RPMI) 1640 (Biochrom, Berlin, Germany) supplemented with 10% FBS, 0.3 mg/mL glutamine, nonessential amino acids, and 1% penicillin-streptomycin. Blood collection was done with a permit from the Landesamt für Gesundheit und Sociales, Berlin, Germany (L 0294/13).

For the reconstitution of mutant and recombinant viruses, EHV-1-BAC DNA was transfected into human embryonic kidney (293T) cells using polyethylenimine (PEI, Polyscience, Niles, IL, USA) [24,25]. Three days later, ED cells were infected with supernatants from transfections.

### 2.2. Plasmids

The complete EHV-1 and EHV-4 *US3* genes were cloned into pcDNA3 (Invitrogen). *US3* genes of EHV-1 and EHV-4 were amplified by polymerase chain reaction (PCR) using primers P1–P4 (Table 1). After purification, the PCR products were digested with the appropriate restriction enzymes and inserted into pcDNA3, generating pcDNA3-US3_1 and pcDNA3-US3_4. The kanamycin-resistance (*kan^R^*) gene was amplified from the pEPkan-S plasmid by PCR using primers P5 and P6 (Table 1), digested with BamHI (New England Biolabs, Ipswich, MA, USA), and cloned into pcDNA3-US3_4 to generate pcDNA3-US3_4kan. Correct amplification and cloning were confirmed by Sanger sequencing (LGC Genomics, Berlin, Germany).

### 2.3. Mutagenesis

Deletion of the whole EHV-1-*US3* gene with the subsequent generation of recombinant EHV-1 harboring US3_4 was done by en passant mutagenesis as described before [26]. Briefly, the *kan^R^* gene was amplified by PCR using primers P7 and P8 (Table 1). After purification, the *kan^R^* amplicon was digested with DpnI (New England Biolabs) and transformed into GS1783 cells containing EHV-1 BACs by electroporation. Kanamycin-resistant colonies were purified and screened by PCR and restriction fragment length polymorphism (RFLP) analyses to identify mutant clones. Positive clones were subjected to a second Red recombination to obtain the final construct, EHV-1∆US3, after removal of the *kan^R^* gene.

The US3_4kan transfer fragment was amplified from the pcDNA3-US3_4kan vector using primers P9 and P10 (Table 1), digested with DpnI, electroporated into GS1783 harboring EHV-1∆US3, and cultured on lysogeny broth (LB) agar with chloramphenicol and kanamycin. Positive clones were isolated and confirmed by PCR and RFLP. Positive recombinant clones were selected and the *kan^R^* gene was excised as shown above.

A point mutation targeting the US3-catalytic active site was engineered by changing the aspartic acid into alanine (US3^D207A^) also by employing two-step Red-mediated recombination. Primers P11–P14 used for mutant (US3^D207A^) and revertant (US3^A207D^) are listed in Table 1. The respective genotypes of mutants and revertants were confirmed by PCR, RFLP, and nucleotide sequencing using primers P15 and P16 (Table 1).

Finally, purified BAC DNA was transfected into 293T cells using PEI. EHV-1 recombinant (mutant) and revertant viruses were further grown on ED cells and stored at −80 °C [27].

### 2.4. Western Blotting

ED cells were infected with different viruses at a multiplicity of infection (MOI) of 1. After 24 h, cell lysates were prepared using radioimmuno-precipitation assay buffer [27]. After lysis, samples were resuspended in loading buffer and heated for 5 min at 95 °C [27]. Proteins were separated by 12% sodium dodecyl sulfate-polyacrylamide gel electrophoresis (SDS-PAGE). Expression of pUS3 was detected with anti-pUS3 polyclonal antibodies, kindly provided by Dr. Dennis J. O’Callaghan (Louisiana State University Health Sciences Center, Shreveport, LA, USA) [28]. As a control for virus infection, cell lysates were blotted and stained with EHV-1 anti-gB small subunit 12D12 monoclonal antibodies (mAb) [29], kindly provided by Dr. Udeni B. R. Balasuriya (Gluck Equine Research Center, University of Kentucky, Lexington, KY, USA). Goat anti-rabbit and anti-mouse labelled with horse radish peroxidase (HRP) were diluted at 1:10,000 in skimmed milk and used as secondary antibodies. Reactive protein bands were visualized by enhanced chemoluminescence (ECL Plus; Amersham, GE Healthcare, Piscataway, NJ, USA).

### 2.5. Virus Growth Assay

To determine virus replication, single-step growth kinetics and plaque diameters were determined as described before [27]. Briefly, confluent ED cells were infected at an MOI of 1 (in the case of EHV-1, EHV-1∆US3, and EHV-1US3_4) or 0.1 (in the case of EHV-1, EHV-1^D207A^, and EHV-1^A207D^) and incubated for 1 h at 37 °C. Cells were washed with PBS, treated with citrate buffer (pH = 3), and supplemented with fresh medium. Supernatant and infected cells were collected separately at the indicated time points and stored at −20 °C. Growth kinetics were determined in three independent experiments. For plaque size assays, ED cells were infected with viruses at an MOI of 0.001. After 1 h of incubation, an overlay was added of IMDM containing 0.5% methylcellulose (Sigma, Darmstadt, Germany) and incubated for 72 h. In three independent experiments, 50 GFP-expressing plaques were photographed for each virus and average plaque diameters were measured using ImageJ software (vl.32j, National Institutes of Health, Bethesda, MD, USA, 2004) [27]. Values were calculated and compared to plaque diameters of parental viruses, which were set to 100%.

### 2.6. Labeling of the Actin Cytoskeleton

ED cells were grown on glass coverslips in a 24-well plate and infected with viruses (MOI = 1) as indicated. After 16 hours, infected cells were fixed with 3.75% paraformaldehyde and permeabilized with 0.2% Triton X-100 (VWR International, Dresden, Germany). After blocking with 2% bovine serum albumin (BSA, AppliChem, Berlin, Germany), polymerized actin of infected cells was detected by staining with phalloidin-Alexa-Fluor-568 (1:40; Invitrogen) for 20 min. After washing three times, coverslips were mounted onto glass slides using Vectashield-with 4′,6-diamidino-2-phenylindole (DAPI; Vector Laboratories, Peterborough, UK). The cells were imaged by immunofluorescence microscopy (Axio imager M1, Zeiss, Jena, Germany) and pictures were taken with an Axiocam CCD camera (Zeiss).

### 2.7. Adhesion Assay

The expression levels of two specific adhesion molecules (very late antigen-4, VLA-4 and lymphocyte function-associated antigen-1, LFA-1) on the surface of PBMC was analyzed by flow cytometry. PBMCs were infected with either parental (EHV-1 or EHV-4) or mutant (EHV-1∆US3) viruses (MOI = 1) or transfected with different expression vectors (pcDNA3, pcDNA3-US3_1 or pcDNA3-US3_4; transfection efficiency was around 40%) using Nucleofector™ (Lonza, Köln, Germany) [30]. Twenty-four hours after infection or transfection, cells were incubated with anti-VLA-4 or anti-LFA-1 antibodies (5 μg/mL; Abcam, Cambridge, UK) for 1 h at 37 °C. Cells were washed three times with PBS and incubated with anti-rabbit Alexa-Fluor-647 (1:1000; Thermo Fisher Scientific, Darmstadt, Germany) for 1 h at 37 °C. Finally, the cells were washed and the expression level of adhesion molecules was quantified among the infected cell population (GFP-expressing cells) using flow cytometry.

### 2.8. Electron Microscopy

ED cells were infected with different viruses (MOI = 1), incubated for 14 h at 37 °C, and fixed with 2.5% (v/v) glutaraldehyde and 2% (w/v) paraformaldehyde in 100 mM cacodylate buffer (pH 7.4) for 30 min at room temperature. Cells were rinsed three times with 100 mM cacodylate buffer, postfixed and stained for 1 h in 1% osmium tetroxide, rinsed three times with distilled water, stained *en bloc* with 0.5% uranyl acetate, dehydrated through an ethanol series and finally embedded using EMBed 812 (EMS, Hatfield, PA, USA). Cells were cut en face and 70–90 nm sections were collected. Sections were counterstained with 4% uranyl acetate followed by lead citrate. All samples were imaged with a Zeiss EM 900 transmission electron microscope equipped with a wide angle CCD camera (TRS-System, Moorenweis, Germany).

### 2.9. Statistical Analysis

Using Prism software (version 5.01, Graphpad, La Jolla, CA, USA), one-way ANOVA, or the Friedman test for repeated measures were applied to test for significance. All data are given as arithmetic means and bars show standard deviations (SD). *p*-values < 0.05 were considered statistically significant.

## 3. Results

### 3.1. Generation of Mutant, Recombinant, and Revertant Viruses

Two-step en passant mutagenesis was used to delete the whole *US3* gene from EHV-1 (EHV-1ΔUS3) and to replace *US3_1* with the *US3_4* gene (EHV-1US3_4) [26]. Furthermore, EHV-1 with a point mutation in the catalytic active site of pUS3 was engineered by changing the aspartic acid into alanine (EHV-1^D207A^). The appropriate revertant viruses, in which the original sequences were restored, were also generated using the same methodology. The correct deletion of *US3_1*, the point mutation and the insertion of *US3_4* were analyzed by PCR, by RFLP by using different restriction enzymes, and by nucleotide sequencing (data not shown).

**Figure 1:**
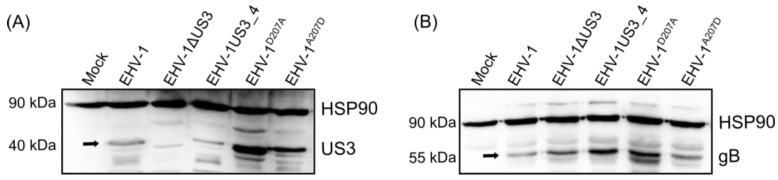
Analysing US3 expression by western blot. Cells were either infected with parental, mutant, recombinant, or revertant viruses. Cell lysates were prepared with radioimmunoprecipitation assay (RIPA) buffer. The pUS3 (**A**) and gB (**B**) proteins were separated by 12% sodium dodecyl sulfate-polyacrylamide gel electrophoresis (SDS-PAGE). The expression of pUS3 and gB (black arrows) were detected with anti-US3 and anti-gB antibodies, respectively. Heat shock protein 90 α/β (HSP90α/β, 1:1000 dilution) was used as a loading control

To determine expression levels of pUS3 by the recombinant viruses, ED cells were infected with the parental, mutant or recombinant viruses and the cell lysates were analyzed by western blotting. The pUS3_1 and the pUS3_4 proteins have a predicted molecular weight of approximately 42 kDa [28]. Parental EHV-1, mutant, recombinant, and revertant viruses expressed pUS3_1 or pUS3_4, respectively, at comparable levels (Figure 1A). As expected, the *US3_1*-deleted mutant did not express pUS3_1 (Figure 1A). It was clear that EHV-1 anti-US3 could react with EHV-4 pUS3; presumably, due to the high similarity between these two proteins (98% at the amino acid level). As a control of virus infection, the expression levels of gB was detected. Proteins with molecular weights of approximately 55 kDa (corresponding to gB small subunit) [29] were detected in all infected cells (Figure 1B). As a loading control, heat shock protein 90 α/β (HSP90α/β) (1:1000 dilution; BioLegend, Fell, Germany) was used (molecular weight: 90 kDa).

### 3.2. Virus Growth Deficit of the EHV-1 US3_1 Mutants Is Rescued by US3_4

**Figure 2:**
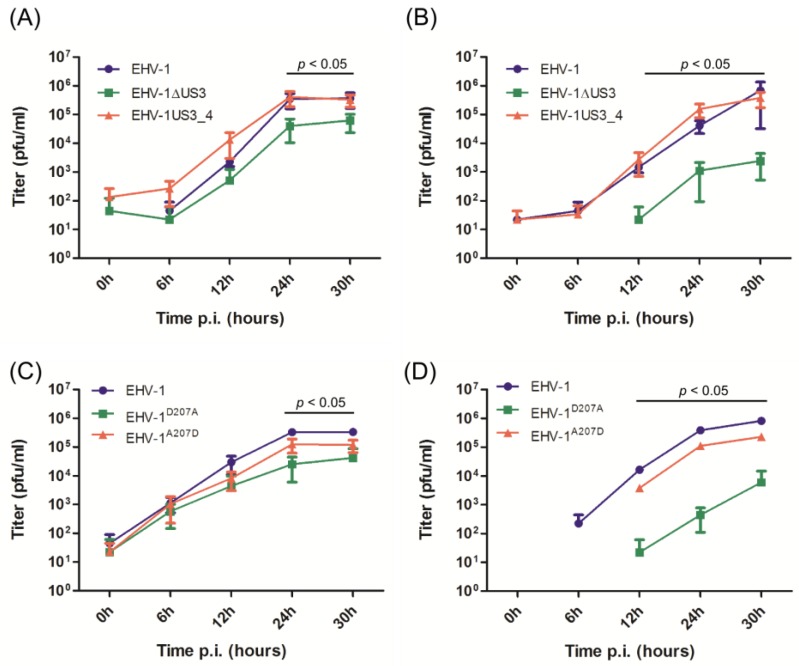
Growth properties of mutant and recombinant viruses. Confluent equine dermal (ED) cells were infected with the different viruses as indicated in the figure. Infected cells (**A** and **C**) and supernatant (**B** and **D**) were collected separately at different time points post-infection (p.i.; 0, 6, 12, 24 and 30 h) and virus titers were determined. The data presented are means ± standard deviations (SD) of three independent experiments. Significance levels (*p* < 0.05) were determined for *US3*-deleted or EHV-1^D207A^ viruses when compared to parental, mutant, recombinant and revertant viruses (Friedman test-Dunn’s multiple comparison test). EHV-1: equine herpesvirus type 1; EHV-1ΔUS3: EHV-1 where *US3* has been deleted; EHV-1US3_4: EHV-1 where *US3_4* replaces *US3_1*; EHV-1^D207A^: EHV-1 with a point mutation in the catalytic active site of pUS3 where aspartic acid was replaced with alanine; EHV-1^A207D^: EHV-1 revertant virus where the original aspartic acid was restored.

**Figure 3:**
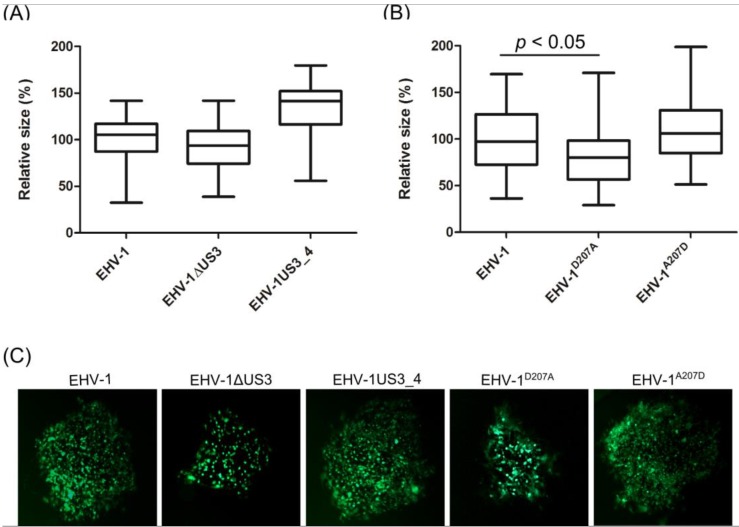
Determination of cell-to-cell spread by plaque size assay. ED cells were infected with EHV-1∆US3 (**A**) or EHV-1^D207A^ (**B**) viruses at a multiplicity of infection (MOI) of 0.001. After 72 h, 50 plaques were measured for each virus. The central line in the box plot indicates the median of the data, while the edges of the box indicate the 25^th^ and 75^th^ percentiles. Extending from the box are whiskers, the top whisker expands to the 95^th^ percentile and the bottom whisker to the 5^th^ percentile. The plaque diameter of parental viruses was set to 100%. A significant reduction (one-way ANOVA; *p* < 0.05) of plaque size for EHV-1^D207A^ was seen when compared to parental, mutant, recombinant or revertant viruses. (**C**) plaques produced by different green fluorescent protein (GFP)-expressing viruses. Pictures were taken using a Zeiss Axiovert fluorescence microscope.

Growth kinetics and plaque sizes were determined in three independent experiments using ED cells. The EHV-1ΔUS3 and EHV-1^D207A^ viruses showed a significant reduction in growth rates at most time points after infection when compared to parental EHV-1, revertant or recombinant EHV-1US3_4 viruses (Figure 2). Similarly, EHV-1ΔUS3 and EHV-1^D207A^ mutants produced smaller plaques (only significant in the case of EHV-1^D207A^) when compared to parental or revertant viruses (Figure 3). On the other hand, US3_4 recombinant viruses induced bigger plaques; however, they were not significant compared to parental viruses (Figure 3). It is worth noting that similar results were obtained with the EHV-1 RacL11 strain [31] (Figure S1). We concluded from the experiments that growth rates and plaque sizes of EHV-1 were significantly affected by the deletion of *US3* or by mutating the catalytic active site, but that the reduced growth rates were restored by the insertion of *US3_4*. This indicated that pUS3 plays an important role in EHV-1 replication and cell-to-cell spread; however, it does not seem to contribute to the difference in pathogenesis of EHV-1 and EHV-4.

### 3.3. Morphological Changes and Actin Stress Fiber Disassembly

**Figure 4:**
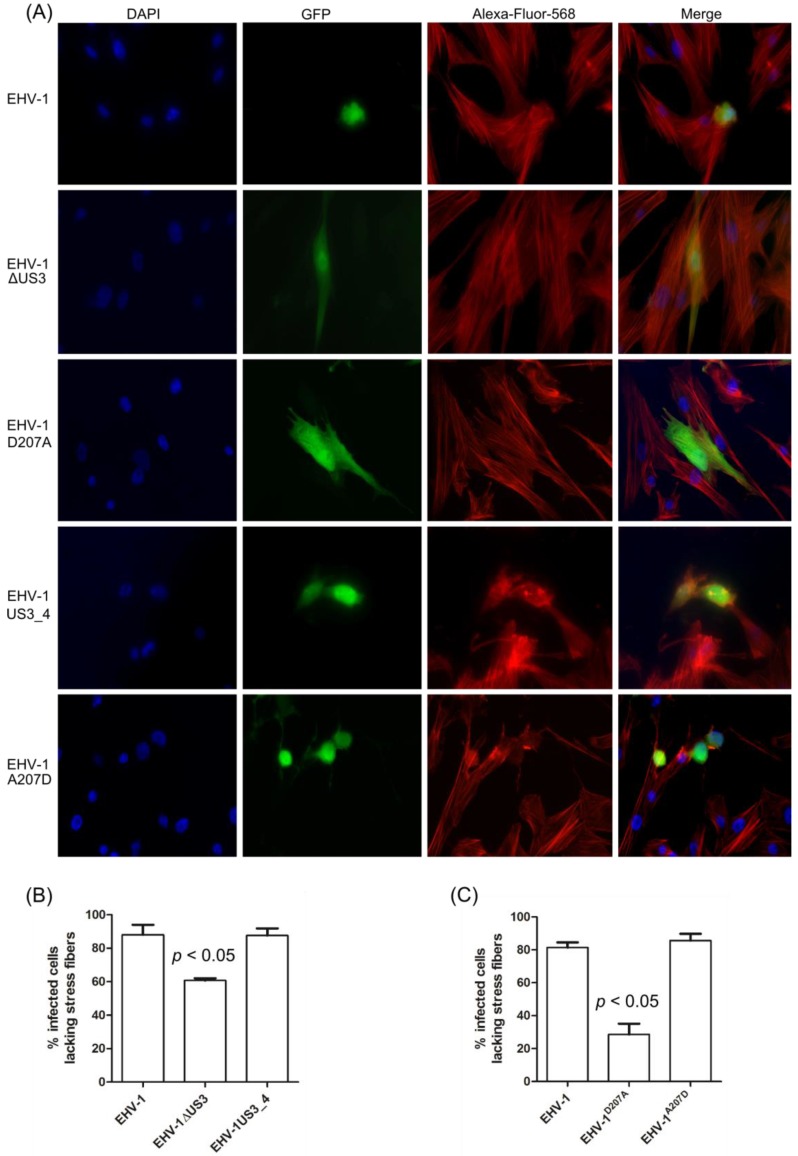
Rearrangement of actin cytoskeleton in infected ED cells. (**A**) ED cells were infected with parental, *US3_1*-deleted, EHV-1^D207A^, *US3_4*-recombinant, or revertant viruses and imaged by immunofluorescence microscopy using a Zeiss Axiovert fluorescence microscope. Pictures were taken with a 63x oil objective. The nucleus was stained with 4′,6-diamidino-2-phenylindole (DAPI, **blue**), the actin cytoskeleton was stained with phalloidin-Alexa 568 (**red**), and the virus-infected cells were visualized through enhanced GFP (eGFP) expression (**green**). (**B** and **C**) A total of 200 infected cells for each virus in three independent experiments were inspected and the percentage of infected cells with or without changes in actin cytoskeleton was calculated. A significant change in actin cytoskeleton rearrangement was detected for either EHV-1ΔUS3 or EHV-1^D207A^ when compared to parental, recombinant, or revertant viruses (one-way ANOVA; *p* < 0.05).

Previous studies with different alphaherpesviruses showed that the pUS3 protein kinase can induce cytoskeletal reorganization in different cell types [6,11,32,33]. ED cells were infected with mutant or recombinant viruses to determine the role of pUS3 in actin microfilament reorganization and cell morphology. EHV-1, the US3_4 recombinant, and revertant viruses induced a significant breakdown of stress fibers and a striking change of cell morphology (Figure 4A). In contrast, infection with the *US3_*1-deleted or EHV-1^D207A^ viruses showed non-significant changes in either cell morphology or reorganization of the actin cytoskeleton after infection (Figure 4A). For quantitative analyses, more than 200 infected cells for each virus (parental, *US3_1*-deleted, EHV-1^D207A^, *US3_4*-recombinant, and the revertant EHV-1^A207D^) in three independent experiments were inspected in a blinded fashion, and the percentage of infected cells with changes in actin cytoskeleton were calculated (Figure 4B,C). The data showed that a mean of 80–90% of cells infected with either the parental, the *US3_4*-recombinant, or the revertant viruses exhibited a disassembly of actin stress fibers. This percent was significantly reduced in cells infected with *US3*-deleted (60%) and EHV-1^D207A^ (30%) viruses (*p* < 0.05; Figure 4B,C). Similar results were also obtained with EHV-1 RacL11strain (Figure S2).

### 3.4. EHV-1 US3 Does Not Affect the Expression of Adhesion Molecules on the Surface of PBMC

In our recent study, we showed that EHV-1∆US3 reduced the ability of infected PBMC to adhere and subsequently transfer virus to EC [16]. We hypothesized that the effect of pUS3 on the actin cytoskeleton might affect the trafficking and, consequently, the expression of adhesion molecules on the cell surface, with the result that it may inhibit virus transfer. We, therefore, infected PBMC isolated from healthy horses with either EHV-1, *US3*-deleted virus, or EHV-4 and determined expression levels of adhesion molecules on the surface of PBMC by flow cytometry.

**Figure 5:**
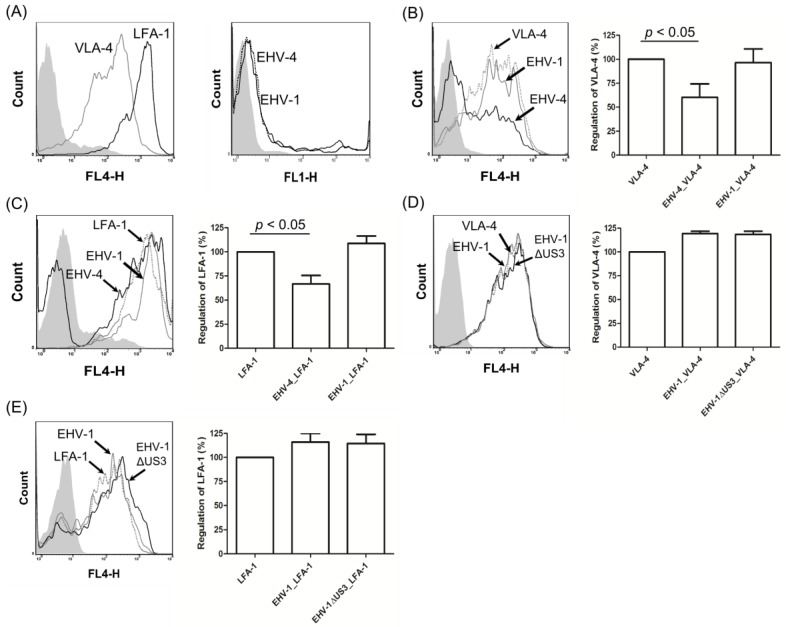
EHV-4 downregulates cell surface adhesion molecules on peripheral blood mononuclear cells (PBMC). (**A**) surface expression of very late antigen-4 (VLA-4) and lymphocyte function-associated antigen-1 (LFA-1) on the surface of mock-infected PBMC and virus infection (**black** line: EHV-1; dotted line: EHV-4) levels of PBMC were determined by flow cytometry. (**B** and **C**) Equine PBMC were either mock-infected or infected with EHV-4 or EHV-1 and subjected to flow cytometric analysis. Histograms and bars of VLA-4 (**B**) and LFA-1 (**C**) expression after detection with the relevant antibodies are shown. Viable cells (10,000) were analyzed for each sample. **Grey** filled: mock-infected cells; dotted line: mock-infected cells with the relevant antibody; **black** line: EHV-4-infected cells; **grey** line: EHV-1-infected cells. For the bars, the expression level of adhesion molecules (VLA-4 or LFA-1) was set to 100%. All data represent the mean ± SD of three independent experiments. A significant downregulation (one-way ANOVA; *p* < 0.05) of VLA-4 and LFA-1 for EHV-4-infected cells was seen when compared to mock- or EHV-1-infected cells. (D and E) PBMC were either mock-infected or infected with EHV-1ΔUS3 and subjected to flow cytometric analysis. Histograms and bars of VLA-4 (**D**) and LFA-1 (**E**) expression are shown. **Grey** filled: mock-infected cells; dotted line: mock-infected cells with the relevant antibody; **grey** line: EHV-1-infected cells; **black** line: EHV-1ΔUS3-infected cells. Data are from one representative experiment out of three. FL1 and FL4: fluorescence detector.

Before investigating the regulation of adhesion molecules on the surface of infected PBMC, the expression of different adhesion molecules in uninfected PBMC was evaluated using flow cytometry. We found that two molecules, VLA-4 and LFA-1, were expressed on 20–50% of PBMC (Figure 5A, left panel). Next, PBMC were either mock-infected or infected with EHV-1 or EHV-4 for 24 h, stained with anti-VLA-4 or anti-LFA-1, and analyzed by flow cytometry. After 24 hours, the percent of infected PBMC reached up to 20% for both viruses (Figure 5A, right panel). Cell surface expression of VLA-4 and LFA-1 was significantly downregulated in EHV-4-infected PBMC (Figure 5B,C), whereas their expression was either not affected or slightly upregulated in the case of EHV-1-infected cells (Figure 5B,C). Infection of PBMC with EHV-1∆US3 did not result in any differences of expression levels of both adhesion molecules (Figure 5D). Furthermore, transfection of PBMC with pcDNA3, pcDNA3-US3_1 or pcDNA3-US3_4 did not affect the level of expression of the molecules either (data not shown).

### 3.5. Accumulation of Enveloped Virions within Perinuclear Vesicles in the Absence of pUS3

**Figure 6:**
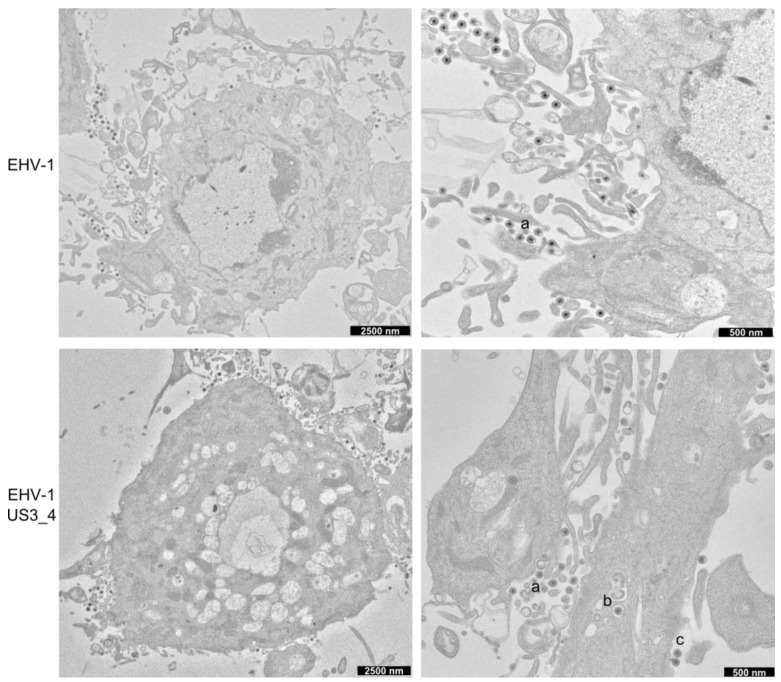
Electron microscopy of ED cells infected with parental and recombinant EHV-1. ED cells were infected for 14 h and analysed by transmission electron microscopy. Cells show different stages of virion maturation, including extracellular virions (**a**), enveloped virions within cytoplasmic vesicles (**b**) and primary enveloped virions in the nucleus (**c**).

**Figure 7:**
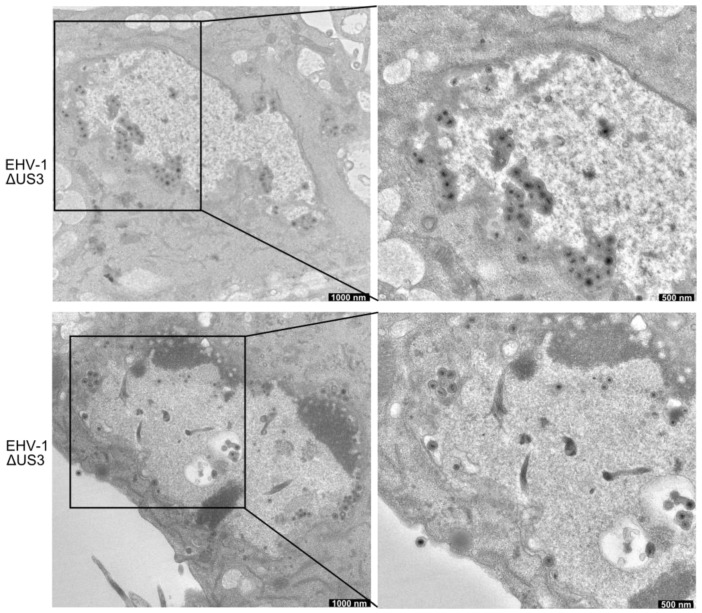
Ultrastructural analysis of ED cells infected with *US3*-deleted EHV-1. ED cells were infected for 14 h and analysed by transmission electron microscopy. Cells show accumulation of enveloped virions in the perinuclear space.

ED cells were infected with parental, *US3_1*-deleted, and *US3_4*-recombinant viruses for 14 h and imaged by transmission electron microscopy. In cells infected with parental EHV-1 or recombinant viruses, numerous extracellular virions released into the extracellular space were readily identifiable (Figure 6). Non-enveloped nucleocapsids and primarily enveloped virions were also seen in the perinuclear space. Other stages of virion maturation, including naked nucleocapsids in the cytoplasm and enveloped virions within cytoplasmic vesicles, were also observed. In contrast, infection with *US3_1*-deleted viruses resulted in the presence of a large number of enveloped virions that accumulated in the perinuclear space within vesicles formed by the inner and outer leaflet of the nuclear membrane (Figure 7). Furthermore, the extracellular space was clearly devoid of enveloped virions.

We next conducted quantitative analyses to investigate the differences in distribution of viral particles in virus-infected cells. Low-magnification electron microscopy (EM) pictures were examined and virus particles were counted for each virus tested in a blinded fashion. The data presented in Table 2 represent the percentages of total particles counted and their distribution as a function of cellular compartment. In cells infected with parental and US3_4-recombinant viruses, between 4 and 6.5% of the total particles counted were found within the perinuclear space. However, in cells infected with *US3*-deleted viruses, most of the virus particles (non-enveloped nucleocapsids and primarily enveloped virions) were trapped in the nucleus either clustered in the perinuclear space (79%) or elsewhere in the nucleus (11%). As described earlier, quantification revealed that more than 93% of the enveloped virus particles were commonly observed on the surface of cells infected with parental or US3_4-recombinant viruses, while in cells infected with *US3*-deleted viruses, the frequency of enveloped extracellular particles was reduced to only 10%.

## 4. Discussion

The US3 serine-threonine protein kinase is conserved among all alphaherpesviruses including EHV-1 [6]. The aim of our study was to assess the roles of pUS3 during EHV-1 infection and to relate our findings to the known functions of pUS3 in other alphaherpesviruses. Furthermore, in our attempt to unravel the functions of viral proteins to understand the striking difference between EHV-1 and EHV-4 regarding replication in vitro and in vivo, we sought to study the functions of pUS3_1 and pUS3-4. We mostly relied on the generation and characterization of mutant viruses where US3 was completely deleted or targeted with point mutations.

Here we show that, like for other alphaherpesviruses, pUS3 is not essential for viral replication. However, plaque size assays and growth kinetics showed that the deletion of *US3* had a modest but significant effect on virus replication and cell-to-cell spread. This loss-of-function was restored after inserting *US3_4* into EHV-1 genome, indicating that the US3_4 protein can efficiently compensate for the function of EHV-1 pUS3 in an EHV-1 background. Similar results on the effect of pUS3 on virus replication were previously obtained after deletion of *US3* in HSV-1, BHV-1, PRV, and MDV [18,19,20,34]. Similarly, targeting the catalytic active site of EHV-1 pUS3 significantly affected virus replication and cell-to-cell spread as shown before in the case of BHV-5, PRV and HSV-2 [33,35,36]. One possible explanation for the stark effect seen is the involvement of the pUS3 protein kinase in the release of primarily enveloped viruses from the nucleus, which leads to the accumulation of virions between the inner and outer nuclear membrane in the absence of the kinase and may consequently result in a less efficient infection of neighboring cells [19]. The EM data presented for EHV-1 and US3 mutant viruses shows that US3 is important for the process of virion de-envelopment at the outer leaflet of the nuclear membrane. However, this important step in virion morphogenesis was restored in the recombinant EHV-1 expressing US3_4.

The *US3*-encoded protein is a serine/threonine kinase that is known to phosphorylate both viral and cellular proteins, but its targets in the case of EHV-1 are still unknown. pUS3 kinase domains are conserved in different alphaherpesviruses including EHV-1 [32]. Previous reports have shown that HSV-1 pUS3 kinase is responsible for building a functional complex between pUL34 and pUL31, which is essential for primary envelopment at the inner leaflet of the nuclear membrane of newly formed nucleocapsids [37,38]. However, in the case of PRV, pUS3 appears to also be involved in the de-envelopment of perinuclear virions by fusion of the primary envelope, which mainly or even exclusively consists of pUL34, with the outer leaflet of the nuclear membrane. Of note, this process is probably not dependent on phosphorylation of pUL34 by pUS3 [18]. Measuring the phosphorylation status of EHV-1 pUL34 and pUL31 in the presence or absence of pUS3 during future experiments will be crucial for determining their role in virus egress from the nucleus.

Besides its role in the de-envelopment process of virions, we demonstrated that the kinase activity of EHV-1 pUS3 also contributes to reorganization of actin, which is deemed important for efficient cell-to-cell spread. As reported for BHV-1, BHV-5, PRV and MDV [20,32,33,39], our data showed that EHV-1 pUS3 induces cytoskeletal modifications leading to breakdown of actin stress fibers. After replacing *US3_1* with *US3_4*, it was evident that the virus-inherent and pUS3-dependent ability to reorganize stress fibers was restored and was indistinguishable from parental virus. The requirement for cytoskeletal modifications has been shown to be different amongst the alphaherpesviruses. While in PRV pUS3 kinase activity seems essential for actin rearrangement, MDV pUS3 does not require kinase activity for the same phenotype [11,20]. In contrast, BHV-5 seems to have kinase-dependent and -independent actin modification functions [33].

Viruses modulate the actin cytoskeleton at almost every step of their replication cycle, starting from entry and ending in egress in order to facilitate spread. One important step during EHV-1 pathogenesis is viral transfer from infected PBMC to endothelial cells, a process that might depend on the upregulation of cellular adhesion molecules in response to virus infection. Recently, we reported that deletion of *US3* reduced the ability of EHV-1-infected PBMC to tether and roll over EC and resulted in a significant reduction of virus transfer from PBMC to EC [16]. We speculated that pUS3-mediated actin rearrangement might be influencing adhesion molecule expression on the surface of infected PBMC [6,40], thereby affecting PBMC-adherence and virus transfer. Our current data show that EHV-4 was able to downregulate adhesion molecules on the surface of infected PBMC, which partly explains the inability of EHV-4-infected PBMC to adhere and subsequently transfer the virus to EC. EHV-1, on the other hand, either did not affect or slightly upregulated adhesion molecules on infected PBMC. Similarly, the expression level of adhesion molecules was not affected after infecting PBMC with EHV-1∆US3, indicating that pUS3-mediated actin reorganization did not affect the expression of the tested adhesion molecules (VLA-4 and LFA-1) on the cell surface. Not only VLA-4 and LFA-1, but also several other adhesion molecules were shown to have a role in mediating firm adhesion of activated PBMC to EC [41,42,43,44,45,46]. We attempted to determine the expression levels of other molecules; however, either the levels of expression were very low or the available commercial antibodies were not reactive with the equine molecules. The possibility that pUS3 affects the expression of other adhesion molecules cannot be excluded. Alternatively, pUS3-induced changes in the actin skeleton may affect the formation and stabilization of clusters of adhesion molecules at the cell surface, thereby altering the adhesive properties of the cell without changing the actual number of adhesion molecules on the cell [47]. Furthermore, other pUS3 activities such as the anti-apoptotic effects may also protect PBMC from clearance before EHV-1 is able to transfer to the EC.

## 5. Conclusions

In conclusion, our report shows that EHV-1 pUS3 is necessary for egress from the nucleus. Furthermore, the serine/threonine protein kinase activity of EHV-1 pUS3 plays an important role in actin cytoskeleton rearrangement. Further investigation of pUS3 kinase activity in infected cells will be conducted to understand the mechanism of its observed role in the infection cycle.

## Figures and Tables

**Table 1:** Oligonucleotide primers used in this study.

No	Primer	Sequence
P1	US3 1 Hind For	attaagcttatggaaaataaacaatgcga
P2	US3 1 EcoR Rev	attgaattcctaaagttgtgcaaacattg
P3	US3 4 Hind For	attaagcttatggaaaataaacaatacga
P4	US3 4 EcoR Rev	attgaattcctacagattcataaacattg
P5	US3 4 kan ins For	gattctagaggggctgcggtaccttcacgaggatgacgacgataagtaggg
P6	US3 4 kan ins Rev	ccctctagaatctgtcgttctataatcaacaaccaattaaccaattctgattag
P7	US3 1 del For	aagccctatagctttataggcacacgcccacggcatcggagatgactaacctgtttctggaggatgacgacgataagtaggg
P8	US3 1 del Rev	gtcgcccacgctgtctcctcccagaaacaggttagtcatctccgatgccgtgggcgtgtgcaaccaattaaccaattctgattag
P9	US3 4 ins into 1 For	gccgtgcgccaagccctatagctttataggcacacgcccacggcatcggaatggaaaataaacaatacga
P10	US3 4 ins into 1 Rev	tttatacaccgtcgcccacgctgtctcctcccagaaacaggttagtcatcctacagattcataaacattg
P11	US3^D207A^ For	tacctgcacgcacagaggatcatccacagagcggtcaagactgaaaatattttcataaacaggatgacgacgataagtaggg
P12	US3^D207A^ Rev	gtttatgaaaatattttcagtcttgaccgctctgtggatgatcctctgtgcgtgcaggtacaaccaattaaccaattctgattag
P13	US3^A207D^ For	tacctgcacgcacagaggatcatccacagagacgtcaagactgaaaatattttcataaacaggatgacgacgataagtaggg
P14	US3^A207D^ Rev	gtttatgaaaatattttcagtcttgacgtctctgtggatgatcctctgtgcgtgcaggtacaaccaattaaccaattctgattag
P15	US3 1 Pre For	ccaactccataaatttcagc
P16	US3 1 Post Rev	ttacagttggtggcactgta

**Table 2:** Virus particles observed in infected equine dermal (ED) cells by electron microscopy (EM).

Virus	% of virus particles			Total Counted (Particles/Cells)
	Nucleus		Extracellular and Cytoplasmic Vesicles	
	Perinuclear area	Intranuclear		
EHV-1	6.5	0	93.5	246/10
EHV-1ΔUS3	79	11	10	203/12
EHV-1US3_4	4	0	96	417/12

EHV-1: equine herpesvirus type 1.

## Supplementary Materials

The following are available online at www.mdpi.com/1999-4915/8/9/275/s1, Figure S1: Characterization of RacL11ΔUS3 virus; Figure S2: Disassembly of actin cytoskeleton in ED cells infected with RacL strain.
