# Supplementary material for: The Role of the Equine Herpesvirus Type 1 (EHV-1) US3-Encoded Protein Kinase in Actin Reorganization and Nuclear Egress
Source: Viruses. 2016 Oct 12;8(10):275. doi: 10.3390/v8100275 (PMC5086611; doi:10.3390/v8100275)
Supplement: Supplementary file 1 [file viruses-08-00275-s001.pdf]

Supplementary Material: The Role of the Equine Herpesvirus Type 1 (EHV-1) US3-Encoded Protein Kinase in Actin Reorganization and Nuclear Egress

Alexandra Proft, Bart Spiesschaert, Satoko Izume, Selina Taferner, Maik J. Lehmann, and Walid Azab

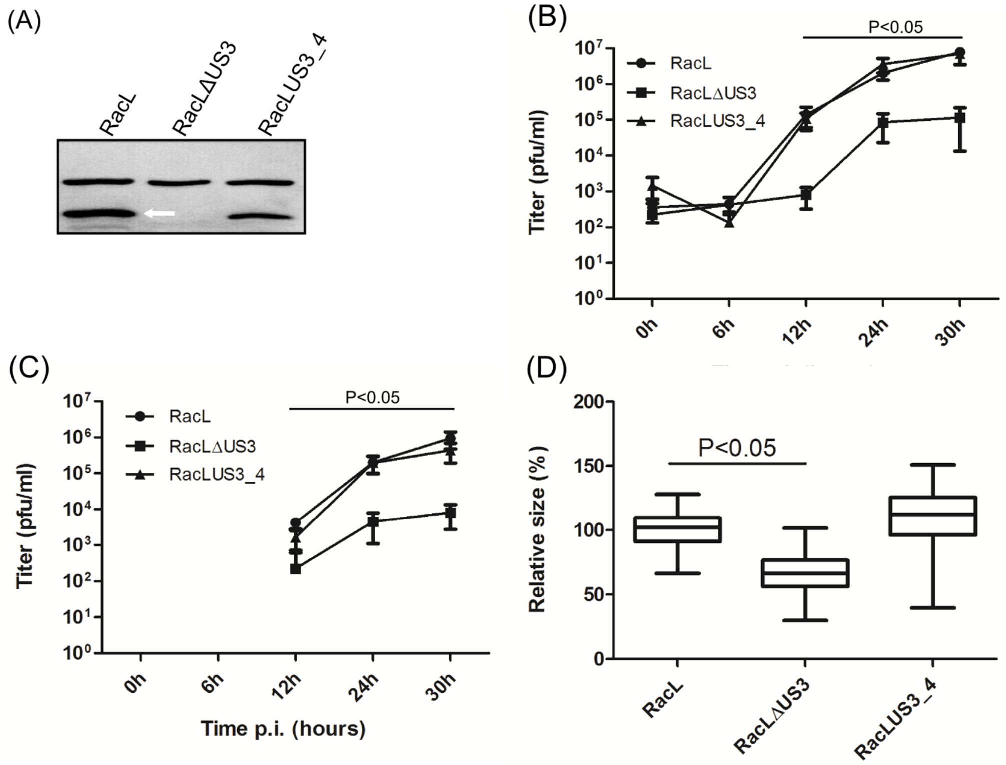

Figure S1. Characterization of RacL11ΔUS3 virus. (**A**) Cells were infected with parental, mutant, or recombinant viruses. Cell lysates were prepared and separated by 12% sodium dodecyl sulfate-polyacrylamide gel electrophoresis (SDS-PAGE). The expression of US3 was detected with anti-US3 polyclonal antibodies. (**B and C**) Growth properties of mutant and recombinant viruses. Confluent ED cells were infected with the different viruses as indicated in the figure. Infected cells (B) and Supernatant (C) were collected separately at different time points and virus titers were determined. The data presented are means ± SD of three independent measurements. Significance levels ($P < 0.05$) were determined for US3-deleted virus when compared to parental and recombinant viruses (Friedman test-Dunn's multiple comparison test). (**D**) Plaque size assay. ED cells were infected at a multiplicity of infection (MOI) of 0.001. After 72 h, 50 plaques were measured for each viruses. Means ± SD of diameters of plaques measured for each virus are shown. The plaque diameter of parental viruses was set to 100%. A significant reduction (one-way ANOVA; $P<0.05$) of plaque size for RacL11ΔUS3 was seen when compared to parental or recombinant viruses.

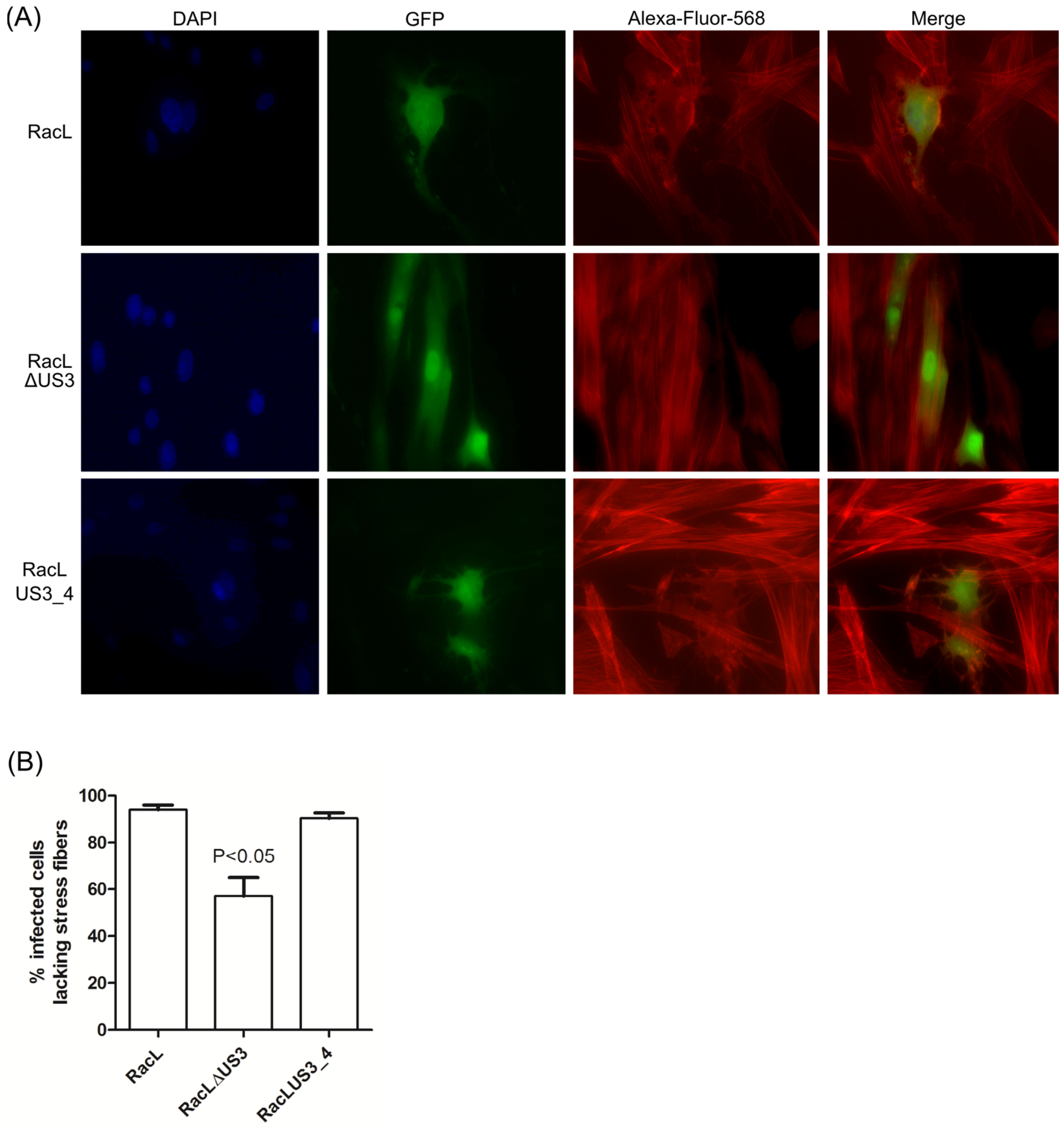

Figure S2. Disassembly of actin cytoskeleton in ED cells infected with RacL strain. (**A**) ED cells were infected with parental, US3_1-deleted, or US3_4-recombinant RacL viruses and imaged by immunofluorescence microscopy using a Zeiss Axiovert fluorescence microscope, and pictures were taken with a 63x oil objective. The nucleus was stained with DAPI (blue), the actin cytoskeleton was stained with phalloidin-Alexa 568 (red), and the virus infected cells were visualised through eGFP expression (green). (**B**) A total of 200 infected cells for each virus in three independent experiments were inspected and the percentage of infected cells with or without changes in actin cytoskeleton was calculated. A significant change in actin cytoskeleton rearrangement was detected for RacLΔUS3 when compared to parental or recombinant viruses (one-way ANOVA; $P<0.05$).

© 2016 by the authors; licensee MDPI, Basel, Switzerland. This article is an open access article distributed under the terms and conditions of the Creative Commons by Attribution (CC-BY) license (http://creativecommons.org/licenses/by/4.0/).
